# Deciphering Latent Health Information in Social Media Using a Mixed-Methods Design

**DOI:** 10.3390/healthcare10112320

**Published:** 2022-11-19

**Authors:** George Shaw, Margaret Zimmerman, Ligia Vasquez-Huot, Amir Karami

**Affiliations:** 1 Department of Public Health Sciences, School of Data Science, University of North Carolina at Charlotte, Charlotte, NC 28223, USA; 2 School of Information Science, Florida State University, Tallahassee, FL 32306, USA; 3 Department of Public Health Sciences, University of North Carolina at Charlotte, Charlotte, NC 28223, USA; 4 Collat School of Business, University of Alabama Birmingham, Birmingham, AL 35233, USA

**Keywords:** sentiment analysis, text-mining, obesity, social media, topic modeling, health communication

## Abstract

Natural language processing techniques have increased the volume and variety of text data that can be analyzed. The aim of this study was to identify the positive and negative topical sentiments among diet, diabetes, exercise, and obesity tweets. Using a sequential explanatory mixed-method design for our analytical framework, we analyzed a data corpus of 1.7 million diet, diabetes, exercise, and obesity (DDEO)-related tweets collected over 12 months. Sentiment analysis and topic modeling were used to analyze the data. The results show that overall, 29% of the tweets were positive, and 17% were negative. Using sentiment analysis and latent Dirichlet allocation (LDA) topic modeling, we analyzed 800 positive and negative DDEO topics. From the 800 LDA topics—after the qualitative and computational removal of incoherent topics—473 topics were characterized as coherent. Obesity was the only query health topic with a higher percentage of negative tweets. The use of social media by public health practitioners should focus not only on the dissemination of health information based on the topics discovered but also consider what they can do for the health consumer as a result of the interaction in digital spaces such as social media. Future studies will benefit from using multiclass sentiment analysis methods associated with other novel topic modeling approaches.

## 1. Introduction

Obesity is a complex health problem and continues to be a major health concern in the United States (U.S.). To encourage physicians to pay more attention to the condition and address the way health insurance companies pay for various treatments, the American Medical Association recently recognized obesity as a disease [1]. There is a need to identify health concerns related to obesity, chronic conditions associated with the disease, and modifiable behavior factors such as proper dieting and increasing physical activity [2,3]. Interviews and surveys are traditional data collection methods for federal and state public health agencies to collect behavioral health data concerning obesity [4,5,6]. While these are well-developed data collection methods [7,8], social media (SM) provides an additional data source to collect behavioral health data, and computational social science provides additional data collection methods [9,10]. Through SM, researchers can effectively and economically collect data about health behaviors and health risk factors. 

People are using SM platforms to disseminate their health experiences and communicate with public health professionals or people with similar health experiences [5,11,12]. This adds a dynamic layer to health information-seeking behavior (HISB) in which such information seeking online is no longer strictly dependent upon static platforms. Within the context of SM, HISB is a layered, complex mechanism across a spectrum of actions and users that can include public health agencies disseminating quality information to fat-shaming conversations on Twitter. While there is value across the spectrum of SM data, many public health agencies are not harnessing the knowledge that resides in these unstructured data and using SM platforms to create meaningful interactions with health consumers [13]. The information shared by users on SM platforms has been harnessed to analyze influenza, E. coli outbreaks, conjunctivitis, and heart disease [14,15,16,17,18]. When looking specifically at Twitter data, initial data collection focused on communicable diseases and began to include noncommunicable diseases as computational methods improved [15,16,19,20]. The improvement of computational science methods is changing how we conduct content analyses aimed at behaviors associated with noncommunicable diseases.

According to Lacy et al. (2015), a content analysis—from its original conceptual understanding—involves the process of categorizing data based on human input to answer a more significant research question surrounding the data [21]. While insightful, traditional content analysis is labor intensive and unfeasible with big data sets, computational approaches expedite this process [22]. Computational content analysis has been used on topics concerning social justice, business, and health [21,23,24]. From a health perspective, the content analysis of user-generated SM data has provided insights into spatial physical activity presence, the prediction of heart disease, and communication of shared user health behaviors [18,25,26].

Prior studies have used social media and various computational approaches to analyze diet, diabetes, exercise, and obesity (DDEO). The authors of [27] sought to identify the influence of social media on public health related to communicated health information using networking modeling. Another study conducted geospatial analysis of tweets to measure happiness, diet, and physical activity [28]. Ref. [2] studied the temporal trends in weight-loss-related posts. These and several additional studies used variations of sentiment analysis, topic modeling, or content analysis to analyze the data. However, these studies did not analyze DDEO topics collectively using SM data. Additionally, there has been limited work using a mixed-methods design to analyze and evaluate DDEO topics [24,29]. 

This research study adds to the breadth of knowledge that uses SM data to analyze health topics but focuses on topic coherence, qualitatively analyzing the relationship among four health topics (diet, diabetes, exercise, and obesity) and distinguishing SM association from HISB. While some public health departments are performing well with disseminating health information, there are opportunities for public health agencies to move beyond basic information dissemination [30]. Many public health agencies lack the support necessary for thoughtful SM engagement. SM has the potential to enhance the communication between individuals and public health agencies [31]. Moreover, understanding the topic discourse that is represented within SM allows public health agencies to be more strategic with information dissemination through this channel of communication [13]. Computational approaches can improve public health department response times to the volume and velocity of data that are generated by SM; refining how quickly we derive knowledge from these data is also harnessed through computational approaches. 

With this study, we attempted to answer the question: What are the positive and negative topical sentiments among diet, diabetes, exercise, and obesity tweets? We attempted to provide a framework for analyzing DDEO health concerns hidden within SM data. The computational experiment is the leading focus of this work; however, secondary to the computational experiment is understanding the topics that are represented with DDEO. This study was designed to be hypothesis generating. Through this research experiment, the two aims of our research question were to: 

(1). Characterize DDEO topics through sentiment analysis and computational topic modeling;

(2). Qualitatively identify the relationships among DDEO topics using the results from the two text-mining procedures.

### 1.1. Background

Obesity prevalence has increased over the past several years with 42.4% of the U.S. population suffering with obesity [7]. Obesity is a well-known risk factor for chronic conditions such as diabetes. People with obesity also experience higher medical costs [1]. Proper dieting and exercising are modifiable lifestyle behaviors that can help with reducing obesity and some of the various chronic conditions associated with it, in particular diabetes [3,31]. While conventional research methodologies have been utilized to gain insight into and characterize behaviors associated with obesity, DDEO data collected from SM require emerging computational methods for their analysis [32]. 

SM has become a fascinating lens through which we can surveil HISB. Never before has there been such a constant stream of residual data to offer insight into the HISB that can be striated so conveniently by population, topic, and time period. In seeking and exchanging health information through SM profiles, it is possible to group users by other public identifiers with some reliability. In this section, the current uses of SM to seek and disseminate health information will be explored, with special attention given to the platform Twitter, as it is the subject of this research.

According to the Pew Research Center, 72% of Americans use at least one SM platform [33]. While uptake is higher among people under 30 than under 50 (90% and 82%, respectively), users between the ages of 50 and 64 are the fastest-growing demographic with 69% using SM as of June 2019 [33]. SM usage is high, above 65%, in all groups when looking at each of the demographics of race, gender, income, education, and community type, such as urban or rural [33]. With such a large proportion of the population using SM, health information has the potential to reach a larger audience as 93 million Americans report that they look for health information online. 

### 1.2. Health Information and SM

The behaviors related to health information seeking and SM are multifarious. SM is often used as a source of social support [33,34,35]. The combination of the private, insular nature of communicating from behind a device and the large community of users with diverse and potentially relevant experience is compelling, particularly with stigmatized issues such as obesity and diabetes [36,37,38]. There is, however, legitimate concern regarding the quality of user-generated health content as SM—including Twitter—has been used by groups and individuals who seek to dissuade others against advice from the medical community [39,40].

Quality assessments of SM information in academic literature are limited, with varying results reported. One study found that half of the health-related tweets analyzed contained false information. In addition, the tweets that did not contain false information were likely to originate from a medical institution [41]. An assessment of user-perceived quality of diabetes-related information on Twitter and Facebook was rated 62 out of a possible 100 [42]. Another study found that while there was high-quality information being disseminated on Twitter, users would need higher literacy skills than the average population’s literacy skills to understand it [43]. An assessment of the usefulness of hashtags for organizing cancer information on Twitter assessed the information to be of high quality but did find that privacy was a great concern regarding sharing medical information in the public domain [44]. 

Another vein of SM research characterizes the types of conversations that users are having on a specific topic [45]. One article explored how humor was used to characterize obesity on Twitter [46]; derogatory jokes were retweeted more than positive ones, and significant attention was given to individual-level instead of societal-level causes for obesity. Mejorva found that fat shaming, or the practice of criticizing a person based on the size of their body, was present in a large share of the discourse happening in the 1.5 million tweets analyzed in their research [38]. Karami and his colleagues explored the various topics present in 4.5 million tweets that discussed diet, diabetes, exercise, and obesity [47]. To demonstrate the relationships between each of the primary topics, subtopics were used to analyze the relationships, and strong correlations were found between exercise and obesity, as well as diabetes and obesity.

### 1.3. Credible SM Information Sources

It is also difficult to differentiate user-generated content from that produced by health professionals. Mejorva’s work incorporating obesity and diabetes discovered that approximately half of the tweets were not affiliated with verifiable, reputable sources [38]. Moreover, tweets from nonreputable sources had a higher likelihood of being retweeted. Another study agreed with this; when assessing retweeting as a metric of reputation on Twitter, it was demonstrated that celebrities and news organizations are more likely to receive a high score than health organizations [48]. A newer study developed a predictive model that assesses the expertise of the user with some success, though vetting for accuracy on SM is an area that warrants considerable concern [49].

Regardless of these issues, there is a legitimate, though not prolific, argument made in scholarship that public health campaigns launched over SM can positively impact users [27,49,50]. SM has been found to be a valuable tool by which to engage the public in order to spread health information [51]. Twitter, in particular, has been utilized to successfully deliver behavioral weight loss interventions and vital diabetes information [28,52,53,54].

## 2. Materials and Methods

### 2.1. Study Design

In order to best address the research aims of this study, we used a sequential explanatory mixed-methods design. Mixed-method approaches in social media research have increased recently. Social media, as a data source, generates data that benefit from the data analysis strengths associated with quantitative and qualitative research. To characterize the topics, we placed more emphasis on the qualitative data [55]. There are an estimated forty mixed-methods research designs [56]. The sequential explanatory mixed-methods design incorporates the quantitative and qualitative findings in order to create more robust results and provide greater depth than either singular analysis would [55,56]. The sequential explanatory design used for this study consists of a quantitative phase that includes data collection and computational analysis, followed by a qualitative phase that incorporates qualitative data analysis to analyze the results from the topic model for evaluation purposes. The quantitative phase for this study incorporates computational steps to collect tweets, clean the data, conduct natural language processing to identify sentiment polarity, and conduct topic modeling. This type of research has been found to be particularly useful in the spectrum of health research [57]. These two phases inform each other, with the qualitative analyzation based on results from the quantitative data; the qualitative phase is used for agreement and the evaluation of the quantitative phase topic model results [56]. Once both the quantitative and qualitative phases have occurred in sequence, the final analysis integrates the findings to enhance the value of the mixed-methods research [57,58]. The following sections outlines the analytical framework) used for this mixed-methods study.

### 2.2. Data Collection and Cleaning

Data used in this study were collected over a three-month period (June 2016–August 2016). These data were extracted from a larger data set that collected data over a 12-month period in 2016 and 2017 and demonstrated that diet (one of the DDEO topics) is important in relation to diet preferences and the political orientation of the state [59]. Using Java programming (Twitter4j) software, the Twitter API was used to amass the data set. Tweets collected were based on their meta-description of English-language, U.S.-based tweets. This method of data collection from Twitter allows you to collect data in real-time; however, this method has several drawbacks: (1) The Twitter API only allows you to stream roughly 10% of the publicly available tweets, (2) specific geo-location information is not always available for every tweet, and (3) there is an absence of observational context to inform the data captured. Therefore, this work did not attempt to analyze the topics according to geographic location. Prior studies have demonstrated dieting behaviors and engagement in physical activity according to geographic location [25]. Health data pertaining to chronic conditions (“diabetes” and “obesity”) and modifiable behaviors associated with chronic health conditions (“diet” and “exercise”) were chosen as query terms. The hashtag and non-hashtag versions of each word in DDEO were used as query terms to search the Twitter API and generate the respective data set for each word. For the query terms, the two versions were used independently of each other during the search process within the Twitter API. The hashtag results and non-hashtag results were merged into one data set, reiterating the need to clean the data. 

The data collection method used for this study involved passive monitoring. Passive monitoring is a low-cost and easy approach to data collection [10,60] (p. 24). Researchers are able to gain insight into the sentiments of users without actively engaging them. Passive monitoring has been used in politics, business, and other health topics [60,61,62,63,64]. Processing of the data collected required cleaning by removing stop words—such as and, of, the—based on a standard list of stop words. Additionally, leading whitespace, numbers, and special characters were removed from the data. This allowed the topic modeling toolkit, used to discover topics, to efficiently identify the topics for analytics purposes.

### 2.3. Sentiment Analysis

Sentiment analysis is a text mining method used to find the polarity (positive, negative, or neutral) in a data corpus. With success, previous studies have used sentiment analysis to detect opinion polarity concerning health topics [65]. This study used the lexicon-based approach to identify the sentiments; the linguistic inquiry and word count (LIWC) tool was used to perform this step of the study. Sentiment analysis was performed on each query term to identify the positive and negative sentiments [66]. The neutral sentiments were not included as part of the analysis. The study focused on sentiment expression for the health topics based on a positive or negative polarity. This approach is often used when capturing positive and negative sentiments using natural language processing techniques [67,68]. Based on this approach, we acquired a total of eight data sets representing the positive and negative polarity for DDEO. 

### 2.4. Topic Modeling

A myriad of health information is communicated in SM spaces. As noted, reputable health care organizations struggle with reaching some intended audiences due to the volume of information disseminated by less credible sources [39,45]. To discover the latent semantic structure and knowledge represented in the data corpora, we conducted text analysis using an unsupervised topic modeling approach. Unsupervised topic modeling is used to discover patterns and describe the knowledge that is represented in unstructured data [68,69]. Using the machine learning for language toolkit (MALLET), the latent Dirichlet allocation (LDA) topic model was used [44,70]. LDA is a common topic-modelling approach, and its performance has been well-documented in other health-related studies involving Twitter data [62,71]. When examining the LDA model the LDA results are two matrices with m words and t topics for a given n of documents. LDA distributes topics over the words P(Wi|Tk) or is expressed as the probability of each word in each topic and the probability of each topic within each document (in this case, tweets) P(Tk|Dj). This allows for a semantically coherent word set [72].

While there is no gold standard for determining the number of topics, several methods have been used to provide objective measures for the optimal number of topics to be analyzed [73]. For this study, we selected 100 topics for each sentiment. Computationally and qualitatively, we determined that this topic number would provide a sufficient representation of the data corpora to successfully perform the analysis for this study [74]. To evaluate the topics identified by the LDA model, we used a qualitative approach. This method does not consider objective analysis with regard to the performance of the model; however, the approach allows for a more in-depth analysis of performance based on topic coherence. Topics were evaluated through the statistical measure of agreement (inter-rater reliability) [72]. 

### 2.5. Topic Evaluation

To evaluate the topics that were identified from the LDA model, Cohen’s kappa was calculated. As previously noted, LDA is an unsupervised topic modeling approach to discovering patterns within a data corpus. Essentially, the model can be trained to cluster together words into topics, which then allows documents with similar topics to be clustered [10]. In this study, we used LDA for the exploratory discovery of topics. Human involvement is necessary for determining themes (topics) and discovering relevant study topics that are difficult to identify when using a topic-modeling method that does not require annotated data [75]. Inter-rater reliability was used to ensure homogeneity in identifying the topics and the stratified relationships among them. If the word in the topic cluster contained a high probability as identified by the model and could be semantically related to another topic, it was identified as being related to another topic. Cohen’s kappa seeks to determine the level of agreement over and above the agreement that is expected through chance [76]. Using this measure, we were able to analyze the topic model results by incorporating a qualitative approach. That is, the topics were evaluated qualitatively with the intent to contextualize the topics. The topic evaluation process involved five steps: 

Step 1: The LIWC tool was used to computationally identify health-related topics and polarity (positive or negative) of the four query terms [47]. 

Step 2: LDA topic modeling was performed on the positive and negative health-related topics as identified through the use of the LIWC tool. Analyzing over one million tweets would have required a substantial amount of human effort. Computationally, LDA performs the process exponentially faster while addressing issues of sparsity related to text mining [77]. 

Step 3: The topic model results were then reviewed by two coders. They identified the topics as being related or unrelated to a DDEO health topic. If they were unrelated to a DDEO health topic, topics were removed, and no additional analyses were conducted on those topics. 

Step 4: After all the non-DDEO-related health topics were removed, the coders were tasked with confirming topic coherence according to their characterization (labeling) as being DDEO related [10,14]. However, unlike the labeling performed in predictive computational studies, the labeling performed in this study was based on analyzing the representative word cluster for each topic. 

Step 5: After the coders characterize the topics independently, they met to discuss disagreements. Once completed, Cohen’s kappa was calculated to measure the agreement after the meeting.

## 3. Results

**Figure 1:**
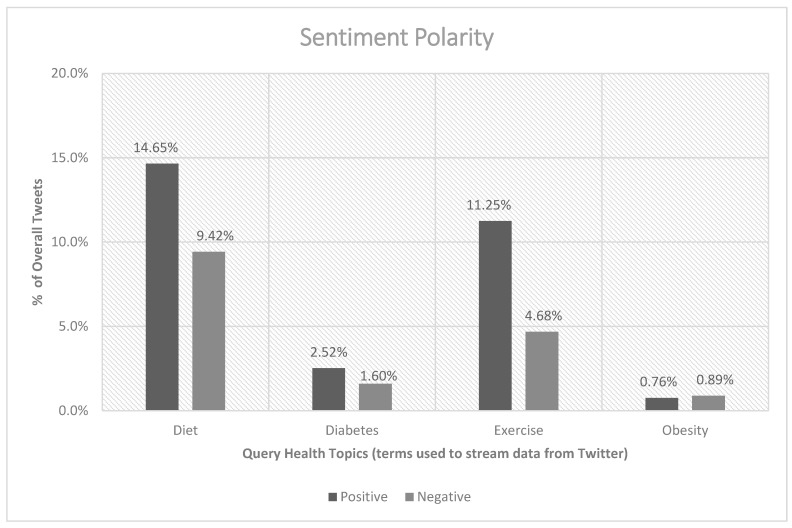
Total sentiment polarity percentages for DDEO.

A total of 15 million tweets represented the data set used in this study. After removing retweets as part of the data cleaning process, the final data corpus consisted of 1.7 million tweets. Our first aim of this research involved characterizing the DDEO health topics using the aforementioned computational approaches. The following sections detail the descriptive statistics of the DDEO topics. When examining the overall positive and negative sentiment compositions of the tweets, 29% were positive and 17% were negative (see Figure 1); the remaining 54% of the tweets were neutral. Among the DDEO topics, the diet data corpus contained the highest number of positive and negative tweets. Positive and negative obesity-related tweets were the least among the DDEO topics. 

Eight hundred topics (100 for each DDEO sentiment) were chosen for the topic analysis. Using the LIWC dimension setting of health on the 800 topics [47], a total of 78 topics were unrelated to their respective health topic (Table 1). Through the qualitative approach, we identified an additional 250 topics that were not DDEO related (Table 1). This approach involved two researchers analyzing the topics according to word clusters. Overall, 59% (473) of the topics were coherent. Obesity was the most-identified topic based on the applied approach; exercise was the least-identified topic (Table 2).

We also examined the prevalence of the remaining topics after step 1 (subsequently removing the 328 unrelated DDEO topics). Diet, diabetes, and obesity showed similar total frequency distribution, with exercise showing the least among the topics. In comparison, negative topics showed a higher prevalence across the topics; exercise was the exception, with a higher distribution across positive topics (Table 2). Our second aim of this research consisted of qualitatively identifying the relationships in DDEO using the results from the sentiment analysis and subsequent LDA model. When stratifying the DDEO topics to evaluate associations based on the topics, obesity had the highest association with the other topics (Table 3). While previous work has utilized statistical approaches to analyze correlations with other topics [47], the qualitative approach allowed for a more nuanced analysis of these topic associations. Although diabetes topics represented 26% of the total number of topics, diabetes had the fewest associations across the other topics based on the content analysis approach used. 

Each topic is represented by T and the numeric value of its positioning among the topics. As noted in Table 4, T1 for positive diet topics represents the first topic (T) from the list of topics (1). Diet-related topics were the most inferable health topic. Diabetes, second to exercise, contained a significant portion of incoherent subtopics. Fifty-eight percent of the topics identified were related to negative sentiments. When analyzing the subtopics, a reoccurring theme we identified was chronic diseases (as noted by T4). The authors of [78] identified chronic disease with a large frequency distribution across negative topics regarding diabetes. When analyzing the subtopics for exercise, many of the positive and negative topics discussed user engagement in physical activity (positive—T4; negative—T36). Additionally, obesity was the only DDEO topic with slightly more negative sentiments than positive sentiments.

### Inter-Rater Reliability and DDEO Relationship

The qualitative content analysis performed on the LDA topic results was also used to establish the reliability of the topics and the relationships among them. Inter-rater reliability demonstrated high reliability with regard to topic coherence of using the LDA topic results for topic analysis regarding DDEO. Additionally, all of the DDEO relationships coded revealed almost perfect agreement between the raters (Table 5). These results indicate the potential of this mixed-methods analytical approach for analyzing topics using unsupervised machine learning. A random sample of coders from a diversified population should be investigated to extend the evidence for and reliability of the analytical approaches we used.

## 4. Discussion

It is difficult to infer the three dominated messages normally found on Twitter—commentaries and opinions, highly personal moment-to moment sentiments and emotions, and informational—through topic model results alone [79]. However, these topics provide insight for health care practitioners who are interested in quickly analyzing large unstructured SM data sets to understand the information being communicated regarding a particular health topic. More importantly, this method uncovers hidden patterns of data (information) that would normally be discarded due to the topics that have a higher frequency distribution within the data set. The following discussion section utilizes the results from the qualitative analytical process and represents the hypothesis generating discussion that would be replicated by health care practitioner’s or public health agencies. Pseudocode was used to increase the anonymity of the tweets analyzed in this study while retaining the original sentiments of the users. However, this process removes the semantical structure of their original communication.

### 4.1. Analyzing the Health Topics Diet

When analyzing positive and negative subtopics for diet, many of the topics appear to reference food or specific diets. As seen in the positive diet topic T4 (Table 3), we infer that the topic is referencing a vegan or vegetarian diet. Several studies have indicated the benefits of a plant-based diet; particularly with reducing people’s risk to chronic conditions like diabetes, cardiovascular disease, and high cholesterol [80,81,82].

Contrary to the health benefits from a plant-based diet, the negative topics associated with diet indicate the consumption of processed food, in-addition to exercising. One twitter reader tweeted “So my dad’s supposed to be on this 30-day diet challenge thing, right? Why did I find a stash of KitKats a few moments ago….” This sentiment is supported by T17. Moreover, T28 also illuminates the emotions that are involved with proper dieting behavior. When we are dealing with negative emotions, impulsive behavior is a mechanism that we use to cope with stress. In some cases, this can lead to overeating and consuming excess calories in a dissociative manner [83].

#### 4.1.1. Diabetes

The positive topics for diabetes covered an array of subtopics like food, spiritual healing, diabetes management, and emotions. As noted in T19, this topic serves as an oxymoron with regard to the diabetes health topic and our interpretation of this topic (sweets). The word cluster for this topic contains foods that are high in sugar with no nutritional value [84]. One user tweeted “my midnight snacks consist of sugar and bagels. Diabetes is what I may have if I continue to eat this way.” Another user says, “Sweat tea from McDonalds is that diabetes in a cup.” Absent from the analysis was the tracking of users over time and the geolocation information. Therefore, we are not able to make inferences about particular geographic regions. However, Nguyen et al. have demonstrated the relationship between healthy food references and economically disadvantaged census tract locations [25].

A latent negative topic inferred from the analytical approach was family history and the relationship with diabetes. One twitter user mentioned the connection between diabetes in their family and current diabetic symptoms. While research does support that people have genetic disposition to the disease, family culture and behavioral factors regarding food consumption plays a role in diabetes prevalence [25,85,86].

#### 4.1.2. Exercise

The sentiment complexity of the exercise topic is captured in the following tweet: “Freedom, exercise, and me time is what my bike has meant to me…more than I can express in words.” Another user tweeted, “On this journey, dieting is so much easier than exercise. I need a personal trainer to get my fitness motivation back suggestions.” For health care practitioners, the latter tweet provides opportunities for user engagement, particularly with improving active participation and two-way communication between SM users and public health agencies. Currently, there is a lack of engagement from public health agencies and health care professionals. Health care practitioners will benefit from creating engaged communities through SM interactions [87,88]. Increased SM engagement also allows health care practitioners to disseminate credible information in spaces that can be dominated by misinformation [89].

Within our positive topics, we also noticed that individuals use Twitter as a digital space to disseminate mobile gaming behavior. Gaming applications are changing how people and researchers view the activities that reflect physical activity [90]. The augmented reality (AR) game—Pokémon Go is an example of mobile gaming behavior that was identified through the topic evaluation (T41). However, this AR application can also lead to unattended accidents due to mobile vehicle distraction and pedestrians lack of awareness in their surroundings [91]. Again, situations like these present opportunities for health care practitioners—public health in particular—to not only disseminate but create engagement with users regarding the drawback of this physical activity behavior.

For this research study, the textual analysis processing task used on the content was completed using n-gram analysis. As a result, this creates an added layer of complexity in the topic analysis process by using the qualitative method. A user tweeted, “I would like to say that the Olympics has inspired me, but it is really due to the fat shaming I expect in California next month.” Based on their tweet, it appears to be some behavioral motivation expressed for exercising, but the remaining portion of their sentiment expresses an alternative motivation factor. The use of another text analysis processing method may have represented these distinguished sentiments better and improved step three of the analytical framework.

#### 4.1.3. Obesity

Positive topic 27 for obesity indicates the potential impact Pokémon Go (Exercise: T41) and other AR gaming can have with addressing childhood obesity. However, there is bleak optimism on AR gaming applications like Pokémon Go and impacting childhood obesity. There are questions regarding the lack of sustainability by these game applications. Physical activity returns to baseline performance after a few weeks [92]. A positive twitter comment supports the link that scientists have made between obesity and 13 types of diseases. These types of comments are identified through topics like T70. Diabetes in men, hypertension, and cholesterol are all chronic conditions that have been associated with obesity [93,94].

A twitter user expressed negative sentiments concerning obesity related conditions: “there is something when you know your life is slowly slipping away because of obesity-related health problems.” For public health departments that focus on oral health, T7 indicates the opportunity to disseminate and engage individuals regarding their oral health. According to the CDC, tooth decay is one of the most prevalent chronic diseases in the United States. Health risk behaviors that consist of drinking and eating foods that are high in sugar, are significant contributors to this problem [95]. State health departments communicating dental health can benefit from the information gathered through SM and the content users disseminate through these platforms [5]. Early SM research involving state health departments and health communication showed low user engagement [13]. However, the use of SM by local or state health departments should focus not only on the dissemination of health information but also consider what the agency can do for the health consumer through those SM interactions.

### 4.2. Implications

This study adds to the breadth of knowledge regarding mixed methods approaches for computational topic discovery. This study also used open-source and low-cost text mining methods to analyze the data. For many public health agencies with limited resources or lack of staff with analytical expertise, these methods can be deployed within their health care setting without significant disruption to current workflows. Additionally, public health practitioners can apply this method to qualitative survey data. Analyzing qualitative survey data using this method may elicit topics that can be important for addressing process measures impacting quality of care for public health care organizations. When considering possible use cases specific to public health practitioners in large cities, this method can be used to possibly identify health concerns through geocoded tweets. This method provides practitioners with a data-driven approach to understanding the needs of the community they serve by using big data to inform decision making [96]. This work also has implications for clinical settings that rely on patient feedback to improve their processes.

From a research perspective, this work adds to the breadth of methodological approaches that seek to discover and interpret the knowledge provided by these data sources regarding DDEO. While this data-driven research is grounded in data science computational methods [10], this work generated a hypothesis that allowed for the application of information-seeking theoretical frameworks. With an effective strategy, this analytical method can be used for other unstructured data sets that are collected by health care practitioners and public health agencies.

### 4.3. Study Limitations

One limitation of this study is that we did not seek to analyze agreement prior to the coders meeting. There were distinct domain differences between the coders related to DDEO, and we expected a weak disagreement between the coders. The lack of context is another drawback of research involving topic modeling. Understanding the relational dynamics of DDEO topic communication on Twitter can be improved by the use and evaluation of other topic model approaches such as the correlated topic model (CTM). CTM allocates the relationships across topics and extends the topical functions of the LDA model [97]. Analyzing the quantity and interaction of DDEO information dissemination among credible sources is an opportunity for additional research. We also did not consider the temporal and spatial data of the tweets. The data used for our study were collected during the summer, and this might have impacted the volume of diet- and exercise-related tweets. Lastly, the sentiment analysis tool utilized in this study calculated sentiment polarity based on the overall sentiment expressed by the tweets. Future studies will benefit from using multiclass sentiment analysis methods associated with machine learning techniques like BERT in conjunction with novel topic modeling approaches.

## 5. Conclusions

People use Twitter and other SM platforms to communicate their health sentiments. These sentiments include health experiences that contain complex semantical structures. Sentiment analysis and topic modeling are effective text mining approaches for topically inferring information from these voluminous data sets. Using these two approaches, we were able to demonstrate the analysis process based on the analytical framework outlined.

When examining the entire composition of the final data corpus (1.7 million tweets), 29% were positive and 17% were negative. Using the computational and qualitative methods, we removed 328 topics that were not DDEO related. However, during the qualitive phase of the topic removal process, we were able to identify three times the number of unrelated DDEO topics. Except for exercise, most of the topics representing DDEO were negative. Diet was the most inferable topic; based on our sample subtopic analysis, food and diets were the most specific topics represented with regard to diet.

Unlike computational approaches that are largely rule-based when classifying topics, the qualitive approach creates challenges when classifying a tweet as DDEO related. Coders infuse their positionality into the process. However, the use of an agreement measure adds an additional method of identifying and evaluating the varying degree coders may have despite a clear coding protocol or equal category proportions [98]. The framework used in this study provides an additional opportunity for transdisciplinary work to be conducted as it relates to DDEO topics. While this framework can be generalized to other social media topics, the nuances involved with examining the word clusters could create concerns regarding the quality of the results. Despite these concerns, additional research with a strong interdisciplinary team is warranted for understanding the potential concerns related to the quality of the results from this analytic framework.

As a digital space, Twitter is a popular SM platform for health communication [99], but many public health practitioners and agencies are using the platform for the one-way dissemination of information. Limited resources and training are needed to conduct this methodology. SM information dissemination should be an initial step in the interaction process to engage SM users and create a relationship beyond the digital space.

## Figures and Tables

**Table 1:** Total number of topics removed using LIWC and inter-rater agreement.

Health Topic	Topics Removed by LIWC (Positive and Negative)	Topics Removed by Coders (Positive and Negative)	Total Topics Removed (Positive and Negative)
Diet	13	54	67
Diabetes	26	65	91
Exercise	11	84	95
Obesity	28	46	73
Total	78	250	328

**Table 2:** Count and frequency distribution of topics after step 2 was completed.

	Positive	Negative	Total
Diet	59 (49%)	62 (51%)	121 (26%)
Diabetes	52 (43%)	70 (57%)	122 (26%)
Exercise	57 (54%)	48 (46%)	105 (22%)
Obesity	58 (46%)	66 (54%)	124 (26%)
Total	226 (48%)	246 (52%)	472

**Table 3:** Stratified distribution of DDEO topic relationships.

	Diet	Diabetes	Exercise	Obesity
Diet		14	28	32
Diabetes	2		3	22
Exercise	27	6		15
Obesity	7	22	5	

**Table 4:** Sample of LDA topics representing each DDEO element (topics were conveniently selected).

	Positive		Negative	
Diet	T1	diet—meat—healthy—fruit—fruits—veggies—vegetables	T17	bad—craving—whataburger—train—break—habit—crossfit
	T4	based—plant—vegan—health benefits—healthy—vegetarian	T18	coke—mcdonald—large—bottle—fridge—hangover—addicted
	T10	diet—diabetes—exercise - blood food—nutrition - sugar	T28	poor—health—problems—obesity emotional—physical—activity
Diabetes	T8	diabetes—care—supplies—insulin—medical—insurance—money	T4	fibrosis—cystic celiac—causing—mellitus—epidemic—disease—endocrine
	T11	diabetes—healing—cancer—god pray—hypertension—energy	T7	loss—weight—diet—exercise—surgery—prediabetes—patient
	T19	Ice—cream—chocolate—love—sugar—coffee—donuts	T20	meat—cancer—antibiotics—hormones—dairy—vegan—diseases
Exercise	T4	exercise—body—stress—yoga—soul—meditation—breathing	T6	weight—lose—diet—fat—eating— food—pills
	T14	fitness—workout—gym—health fitfam—training—cardio	T25	stress—depression—anxiety—helps endorphins—brain—mood
	T41	exercise—pokemon—playing pokemongo—people—walk—game	T36	hate—running—gym—worst—working—kind—stupid
Obesity	T16	obesity—activity—physical—social—reduce—active—fitness	T7	poor—diabetes—dental—warning soda—consumption—health
	T27	obesity—pokemon—childhood epidemic—america—pokemongo walking	T16	poka—obesity—bmi—time—game proportional—complication
	T70	diabetes—obesity—cancer disease—cholesterol—hypertension—insulin	T44	syrup—corn—obesity—promoted fructose—markets—household

**Table 5:** Cohen’s kappa agreement for each topic based on DDEO coherence agreement and identification of topic relationships.

	Positive		Negative	
	Inter-Rater Reliability		Inter-Rater Reliability	
Topic	Topic Coherence	DDEO Topic Relation	Topic Coherence	DDEO Topic Relation
Diet	1.000	1.000	0.955	1.000
Diabetes	0.958	1.000	0.969	1.000
Exercise	0.979	0.921	0.978	1.000
Obesity	0.885	1.000	1.000	0.969

## Data Availability

Data available upon request.
